# Zika Virus: A Tale of Two Lineages

**DOI:** 10.3390/pathogens14111151

**Published:** 2025-11-12

**Authors:** Inès Bribes, Sébastien Nisole

**Affiliations:** Institut de Recherche en Infectiologie de Montpellier (IRIM), Université de Montpellier, CNRS, 34090 Montpellier, France

**Keywords:** Zika virus, viral evolution, arbovirus emergence, African lineage, Asian lineage, neurovirulence, innate immunity, vector competence

## Abstract

Zika virus (ZIKV) was first identified in Africa in the mid-20th century and circulated for decades with limited and often unnoticed human cases. This situation changed with the emergence of the Asian lineage, responsible for large outbreaks in the Pacific and the Americas and for severe complications such as Guillain–Barré syndrome and Congenital Zika Syndrome (CZS). In contrast, the African lineage, although frequently more efficient in replication, cytopathogenicity, and mosquito transmission in experimental systems, has not been linked to comparable epidemics or congenital disease clusters. This review summarizes current knowledge on the differences between African and Asian lineages at the molecular, cellular, and epidemiological levels. It highlights how genetic variation interacts with host immunity, ecological factors, and human activity to shape epidemic potential. Understanding these interactions is essential for anticipating future outbreaks and for improving strategies to mitigate the impact of emerging arboviruses.

## 1. Introduction

Vector-borne diseases are an increasing global health concern, with their distribution expanding under the combined pressures of climate change, urbanization, and intensified international travel [1]. Among the most concerning vectors are mosquitoes of the genus *Aedes*. The Asian tiger mosquito (*Aedes albopictus*), which shows a great tolerance to colder climates, has successfully colonized temperate regions across several continents, while *Aedes aegypti* is predicted to expand into temperate areas in the coming decades [1,2]. These shifts are expected to increase the risk of mosquito-borne viruses, particularly of *Orthoflaviviruses* such as dengue virus (DENV, *Orthoflavivirus denguei*), yellow fever virus (YFV, *Orthoflavivirus flavi*) or Zika virus (ZIKV, *Orthoflavivirus zikaense*), in areas that were previously unaffected, including Southern and Eastern Europe, North America, and temperate regions of Asia [3]. According to the World Health Organization (WHO), over 80% of the world’s population is at risk of at least one vector-borne disease, and more than half face the threat of multiple infections [4]. Beyond their epidemic potential, flaviviruses are a major public health challenge due to their severe clinical outcomes, ranging from dengue hemorrhagic fever, yellow fever–associated hepatitis, and neurological complications including encephalitis, paralysis, and cognitive disorders caused by viruses like West Nile virus (WNV, *Orthoflavivirus nilense*), Japanese encephalitis virus (JEV, *Orthoflavivirus japonicum*), and ZIKV [5]. Understanding the mechanisms that underlie orthoflavivirus emergence and identifying the factors that shape their epidemic potential is critical for anticipating and mitigating future public health threats.

ZIKV exemplifies the dynamic nature of orthoflavivirus emergence. First identified in 1947 in Uganda [6], ZIKV was long regarded as a relatively benign pathogen, causing only sporadic and mild infections in Africa and Asia [7]. This perception changed dramatically in the late 2000s and 2010s, when ZIKV spread across the Pacific and subsequently the Americas, triggering large-scale outbreaks associated with severe neurological diseases. These included Guillain–Barré syndrome and, most notably, Congenital Zika Syndrome (CZS), in which maternal infection caused profound developmental abnormalities in infants [7,8,9]. The scale and severity of these epidemics prompted the WHO to declare ZIKV a Public Health Emergency of International Concern (PHEIC) in 2016 [10].

One of the most intriguing aspects of ZIKV biology is the existence of two major genetic lineages: African and Asian [11]. Despite their shared ancestry, only the Asian lineage has been associated with widespread outbreaks and severe disease outcomes, whereas the African lineage has remained largely restricted to sporadic, localized cases. This divergence raises a central question: what molecular factors enable a lineage to evolve from a neglected pathogen into an epidemic-prone and neurotropic virus? In this review, we summarize the current knowledge on the phenotypic and molecular differences identified between African and Asian ZIKV lineages, with a particular focus on the evolutionary changes that may underpin ZIKV’s emergence and public health impact.

## 2. Methodology of the Review—Literature Search Strategy

A comprehensive literature search was conducted in the PubMed database up to June 2025 using combinations of the following keywords: “Zika virus”, “lineages”, “African”, “Asian”, “pathogenicity”, and “evolution”. Priority was given to articles reporting experimental or epidemiological comparisons between ZIKV lineages. Additional relevant studies were identified through the bibliographies of selected articles. Non-peer-reviewed articles, case reports, and publications not available in English were excluded. We acknowledge that, due to space limitations, some valuable contributions could not be cited.

## 3. Zika Virus Biology

### 3.1. Epidemiology

**Figure 1:**
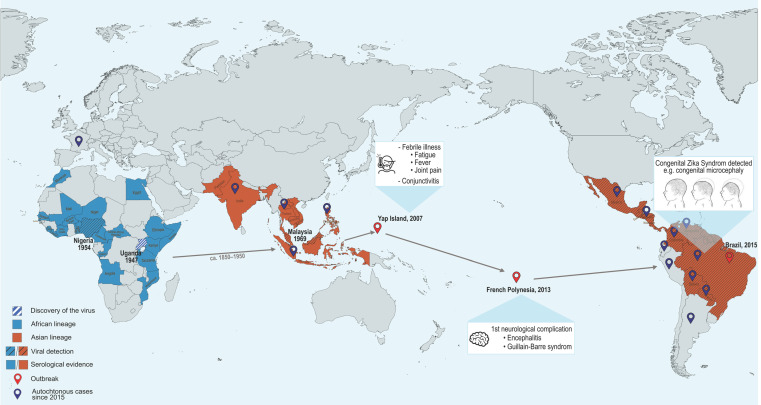
**Epidemiological overview of Zika virus emergence.** The map illustrates the geographic spread of Zika virus from its initial discovery in Uganda in 1947 to the present. It also summarizes the evolution of associated clinical manifestations and the current epidemiological situation following the 2015 outbreak. Created using MapChart (available at https://www.mapchart.net).

ZIKV was first isolated in 1947 from a rhesus macaque in Uganda’s Zika forest during yellow fever surveillance, and subsequently from *Aedes* mosquitoes in the same area [6] (Figure 1). It was later isolated in Asia in 1969 from *Aedes aegypti* mosquitoes [12]. The first isolation of the virus in humans occurred in Nigeria in 1954 [13], while serological studies suggested silent circulation among African populations [7,14]. Between 1947 and 2007, only sporadic cases were reported in Africa (including Uganda, Nigeria and Tanzania…) and Asia (including the Philippines and Malaysia…), all presenting mild symptoms and attracting limited scientific attention [7,15].

The first outbreak was reported on Yap Island (Federated States of Micronesia) in 2007, with over 100 confirmed or symptomatic cases and an estimated 73% residents infected [16] (Figure 1). Symptoms included fever, joint pain, rash, and conjunctivitis, the latter distinguishing ZIKV from DENV infection and leading to its identification as the causative agent [17].

In late 2013, a second outbreak occurred across all 67 islands of French Polynesia and spread to New Caledonia, the Cook Islands, and Easter Island (Chile) [18]. Around 30,000 cases were estimated, representing 11.5% of the population including asymptomatic infections [19,20]. For the first time, neurological complications were reported, notably Guillain-Barré syndrome, an autoimmune attack against the peripheral nervous system [21,22] (Figure 1).

In 2015, ZIKV reached the Americas, causing a large-scale epidemic beginning in Brazil, where up to 1.3 million suspected cases were estimated [23]. By the end of the year, the Pan American Health Organization and WHO linked ZIKV to congenital microcephaly and other neurological complications in infants from infected pregnancies, later defined as CZS [24]. Soon after, ZIKV’s designation as a PHEIC underscored its transition from a relatively neglected virus associated with mild, sporadic infections to a neurotropic virus with epidemic potential [10]. The virus then spread to Colombia, Guatemala, Mexico, and the Caribbean, while Cape Verde reported approximately 5000 suspected cases, marking ZIKV’s reemergence in Africa via an Asian lineage [25,26] (Figure 1).

Although no large outbreaks have occurred since 2016, ZIKV continues to circulate in multiple regions [27]. The expansion of *Aedes* mosquitoes in Europe raises concerns about future epidemics, particularly in immunologically naïve populations [28,29] (Figure 1).

### 3.2. Virion Structure and Genome Organization

**Figure 2:**
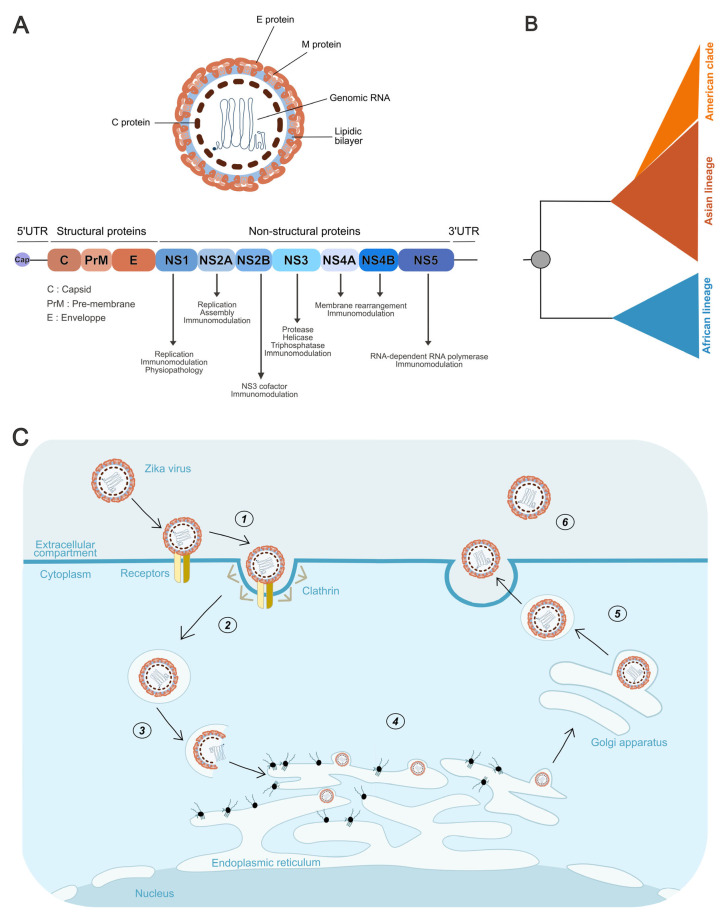
**Genomic Organization, Phylogeny, and Life Cycle of Zika Virus.** (**A**) Schematic representation of the Zika virus particle and its genome organization. The single open reading frame encodes a polyprotein, which is processed into structural proteins (C, prM/M, E) and nonstructural proteins (NS1–NS5). Annotations indicate the known functions of each nonstructural protein. (**B**) Schematic phylogenetic tree of Zika virus illustrating the two main lineages, African and Asian, with the American clade forming a distinct branch within the Asian lineage. This schematic representation is based on previously published phylogenetic analyses [8,9,56,57,58,59,64]. (**C**) Schematic representation of the Zika virus replication cycle. (1) The Zika virus attaches to specific cellular receptors on the host cell plasma membrane. (2) Viral entry occurs through clathrin-mediated endocytosis. (3) Fusion of the viral envelope with the endosomal membrane leads to the release of viral RNA into the cytoplasm. (4) The positive-sense RNA genome is translated, initiating the formation of viral replication complexes within the endoplasmic reticulum (ER) membrane, where genome replication and virion assembly take place. (5) Immature virions undergo maturation in the Golgi apparatus through prM cleavage by furin. (6) Mature virions are subsequently released from the host cell via exocytosis. Created using Inkscape 1.4.2.

ZIKV shares the canonical architecture of all Orthoflavivirus virions. The positive-sense RNA genome is encapsidated by capsid (C) proteins, forming the nucleocapsid (NC), which is surrounded by a host-derived lipid envelope (Figure 2A). Embedded within this bilayer are the surface glycoproteins E (envelope) and prM (pre-membrane), the latter being cleaved into M during the maturation process. Virions measure approximately 50 nm in diameter [30]. The genome is a single-stranded, positive-sense RNA of ~10.8 kb encoding the main long open reading frame (ORF) that is translated into the viral polyprotein. This ORF is flanked by a canonical 2′O-methylated cap at the 5′ end [31] and by complex secondary and tertiary structures at the 3′ end, which lacks a poly(A) tail [30] (Figure 2A).

A short upstream open reading frame (uORF), recently identified by ribosome profiling (Ribo-Seq), lies upstream of the main ORF. Its expression, likely arising from ribosomal leaky scanning, can account for up to 4% of the expression of the main ORF and has been shown to modulate ZIKV virulence and replication efficiency [32,33,34].

Beyond the uORF, the untranslated regions (UTRs) at both 5′ and 3′ ends exert tight control over viral translation, replication, and genome stability. The 5′UTR, approximately 100 nucleotides in length, contains sequence motifs and structured RNA elements that play a crucial role in replication: they enable viral circularization as well as NS5 binding via its methyltransferase (MTase) domain, which allows the RNA-dependent RNA polymerase (RdRp) domain to properly position at the 3′ end and initiate negative-strand synthesis [35]. The 3′UTR is longer and contains sequences complementary to the 5′UTR, thereby enabling circularization, and harbors structured elements involved in replication, translation [36,37], and resistance to interferon-stimulated genes (ISGs) [38]. Mutations within this region can enhance replication in the mosquito vector, underscoring its role in host adaptation [37,39]. Moreover, the 3′UTR generates flaviviral subgenomic RNAs (sfRNAs) upon stalling of the host 5′–3′ exoribonuclease XRN1 at stable RNA structures, leading to incomplete genome degradation [40]. sfRNAs fulfill multiple functions, including inhibition of RNA interference in insects, modulation of type I interferon (IFN-I) response, and antagonism of cellular antiviral factors [41].

The main ORF encodes a polyprotein processed by viral and host proteases. This ORF is organized into upstream coding regions for structural proteins and downstream regions for nonstructural proteins (Figure 2A).

Structural proteins include: C, which dimerizes around the genome to form the nucleocapsid [30] and also contributes to genome circularization [42]; prM, which functions initially as a chaperone for E folding and assembly and later associates with E dimers to form spike-like structures that flatten upon maturation; and the E protein, which forms dimers and contains both the receptor-binding site and the fusion peptide required for entry [30].

The nonstructural proteins are encoded downstream and fulfill essential functions in replication, immune evasion, and pathogenesis. NS1 has both intracellular and extracellular functions. Within infected cells, it associates with endoplasmic reticulum membranes and contributes to the assembly of the viral replication complex [43]. It is also expressed at the cell surface and secreted as a hexamer, which is strongly immunogenic and inhibits complement activation [44,45]. Circulating NS1 binds endothelial cells across multiple organs (lungs, dermis, brain, liver), thereby altering vascular permeability and promoting viral dissemination [46]. Importantly, NS1 is a determinant of neuroinvasion, as glycosylation site changes can increase brain entry [47,48], and specific mutations have been linked to ZIKV-associated microcephaly [49,50]. NS1 is also secreted in the mosquito vector, where mutations enhance infectivity in Aedes mosquitoes [51].

NS2 is divided into NS2A and NS2B. While the precise role of NS2A remains unclear, it participates in viral assembly [52] and was recently shown to inhibit selective endoplasmic reticulum autophagy (reticulophagy), thereby favoring replication and contributing to pathogenesis [53]. NS2B is well defined as a cofactor for NS3, essential for polyprotein cleavage [43]. NS3 is multifunctional: its N-terminal domain encodes a serine protease, essential for polyprotein cleavage in cooperation with NS2B, while its C-terminal domain functions as a helicase, unwinding dsRNA intermediates, and as a 5′-RNA triphosphatase, contributing to RNA capping in coordination with NS5 [43]. NS4 includes NS4A and NS4B. NS4A bridges NS1 to the NS3/NS5 replication complex and is indispensable for replication. Both NS4A and NS4B colocalize and mediate endoplasmic reticulum membrane rearrangements required for replication complex formation [43,54]. Finally, NS5 is the RdRp and possesses methyltransferase activity required for RNA capping, acting in concert with NS3 during positive-strand synthesis [43] (Figure 2A). In addition to their replication functions, nonstructural proteins also play major roles in suppressing innate immune defenses [55] (Figure 2A).

### 3.3. Phylogeny of Zika Lineages

Phylogenetic analyses of ZIKV have identified two major lineages, African and Asian, both belonging to the same serotype [11] (Figure 2B). Comparative studies have revealed 50–100 amino acid differences across the coding region, corresponding to ~12% divergence, along with additional variations in E protein glycosylation that influence viral properties such as neurovirulence [8,56,57].

The African lineage is believed to have emerged well before the mid-20th century, with estimates varying from the late 18th to the early 20th century [57,58,59]. Depending on the genomic region examined, it can be resolved into either two clusters (MR766/West African and Nigerian) or a single group [57,59,60]. Although its origins lie in Africa, this lineage has also been detected in sylvatic transmission cycles in Brazil, highlighting its broader ecological range [61,62].

The Asian lineage is thought to have arisen from an African ancestor, likely during the 1950s [8,57,58,63], following a single introduction that subsequently gave rise to strains responsible for recent outbreaks [8,56,57,58]. However, one molecular clock analysis suggests an earlier divergence, around 190 years ago [59]. All Asian strains trace back to a common ancestor with the P6-740 strain, isolated in Malaysia in 1966. Compared with African viruses, strains derived from P6-740 carry 26 amino acid substitutions in the polyprotein, while epidemic strains circulating since 2013 harbor eight additional conserved mutations, except for a variable site in NS5 (T2634M/V) [56].

Within the Asian lineage, an “American” clade encompasses strains from Brazil and from other countries in the Americas, including the Caribbean, in contrast to the “Pacific” clade [9,58,64]. These viruses share over 99% genomic identity with strains from French Polynesia, reflecting a founder effect from the 2013 epidemic and its subsequent dissemination across the Americas [65,66]. Following introduction into the Americas, ZIKV diversified into four sub-lineages, likely arising from a limited number of importation events [67] (Figure 2B).

The evolutionary rate of ZIKV is estimated at 10^−3^ to 10^−4^ substitutions per site per year, comparable to other orthoflaviviruses [57,59]. Evolution is dominated by purifying selection, with synonymous substitutions accumulating while nonsynonymous changes remain rare, suggesting that ZIKV is already well adapted to its ecological niche [57,59].

Recombination events have been reported, particularly in the E and NS5 genes of the African lineage [57,59,60] and between the Asian lineage and Spondweni virus in NS2B [68]. Although recombination is generally rare in orthoflaviviruses compared with other RNA viruses, experimental evidence confirms that it can occur [69,70,71,72].

Defective viral genomes (DVGs) generated through recombination have also been identified in vitro, where they appear to attenuate viral replication and pathogenicity. Their relevance in natural settings and potential role in ZIKV evolution remain unclear [73].

### 3.4. Viral Cycle

The ZIKV life cycle begins with clathrin-mediated endocytosis following attachment to host cells (Figure 2C). In mammalian hosts, virions first engage low-affinity attachment factors such as glycosaminoglycans, which concentrate particles at the cell surface [74,75]. They then interact with specific receptors including C-type lectins (e.g., DC-SIGN) [76,77,78], GRP78/BiP [79], CD14 [80], and TAM receptors (Tyro3, AXL, Mer) [81]. Viral E protein binding directs particles toward clathrin-rich membrane regions, where invagination and dynamin-mediated scission generate endosomes [82].

ZIKV can also exploit antibody-dependent enhancement (ADE), a mechanism originally described for DENV, where cross-reactive but non-neutralizing antibodies facilitate uptake into Fc receptor–bearing immune cells, thereby enhancing infection [83,84].

After internalization, endosomal acidification triggers E protein rearrangement into fusion-competent trimers that mediate membrane fusion and nucleocapsid release into the cytosol. The capsid then dissociates, liberating the genome, which is translated at the ER into a single polyprotein [30] (Figure 2C). This polyprotein is processed by viral (NS3) and host proteases, generating structural and non-structural proteins. Non-structural proteins remodel ER membranes into replication organelles (“viral factories”), which shield viral RNA synthesis. Genome circularization initiates negative-strand synthesis by NS5, followed by production of positive-strand RNAs. NS1 contributes to replication complex stabilization and ER remodeling, while NS4A and NS4B mediate further membrane rearrangements. NS3 also functions as a helicase and RNA triphosphatase, and together with NS5 ensures proper RNA capping and methylation [35,43,85,86].

Assembly occurs at the ER, where the nucleocapsid, composed of RNA and C protein, buds into the lumen at sites enriched in prM and E (Figure 2C). This generates immature particles, which mature during passage through the trans-Golgi network. Acidification induces conformational changes in prM and E, followed by furin-mediated cleavage of prM, producing the structural rearrangements that yield infectious virions [30] (Figure 2C). Mature virions are released from the cell through exocytosis (Figure 2C). However, a proportion of particles remains immature and lacks full infectivity. Despite being non-infectious on their own, these immature virions can still be internalized by host cells through ADE [87,88].

## 4. In Vitro Differences Between Lineages

### 4.1. Viral Replication

Comparative in vitro studies highlight lineage-specific replication dynamics of ZIKV (Table 1). Overall, the African lineage tends to replicate more rapidly and efficiently than the Asian lineage across diverse cell types, although some exceptions exist.

In immortalized cell lines of mammalian and insect origin, African strains generally exhibit higher replication rates (Table 1). This pattern has been observed in human HEK-293 and A549 cells, avian DEF cells, rabbit RK-13 cells, and multiple mosquito-derived cell lines, including *Aedes* (C7/10, RML-12, AP-61, C6/36), *Anopheles gambiae*, and *Phlebotomus papatasi*. An exception to this trend was noted in Vero cells, of simian origin where no significant difference in replication efficiency was detected [89,90,91,92]. In a complementary study, Ou et al. (2021) compared two Asian strains and one African isolate in Aag2 cells, derived from *Aedes aegypti* embryos, and observed the reverse pattern: the African isolate replicated more slowly [92]. However, the authors suggested that this result was likely influenced by the pronounced cytopathic effect induced by the African strain, which could have affected replication measurements [92]. This characteristic will be further discussed in the next section.

In more physiologically relevant models, particularly dendritic cells (DCs), which are among the first targets of cutaneous infection, African strains also exhibit faster replication kinetics and higher viral loads [93] (Table 1). In contrast, Österlund and colleagues reported opposite findings: under their conditions, Asian strains replicated better and elicited a stronger antiviral response in DCs. However, their study included only one African strain, and they observed comparable replication between the two lineages in macrophages [94].

Enhanced replication of African strains has been consistently reported in neural models, including neuroblastomas, glioblastomas, neural progenitors, and primary human neurons and astrocytes [95,96,97,98]. Superior replication has also been noted in human pericytes, as well as across the blood–brain barrier (BBB) and the retinal barrier [99,100].

With regard to ZIKV effects on the fetus, viral replication was assessed in fetal pericytes. This model supports the trend, as African ZIKV strains displayed faster replication, although viral titers dropped at 8 days post-infection, while Asian strains persisted up to 14 days [101]. In trophoblasts derived from embryonic stem cells, Megan A. Sheridan and colleagues published two studies assessing viral replication. The first reported stronger infection and higher virion production for the African strain tested [102], whereas the second, which included two additional strains of each lineage, showed similar replication rates [100]. However, severe placental lysis was observed only following infection with African strains [102,103].

Finally, organotypic brain slice cultures from embryonic mice revealed initially higher replication by African strains, with a pandemic Puerto Rican strain later reaching comparable levels, unlike other Asian strains [104].

Together, these data suggest that the African ZIKV lineage generally displays superior replication kinetics and magnitude across multiple cellular models (Table 1).

### 4.2. Cytopathogenicity

Overall, the African lineage of ZIKV exhibits greater cytopathogenicity than the Asian lineage (Table 1). In immortalized cell lines (HEK-293, Vero), African strains induced stronger cellular damage, observed using plaque assays [89]. Similar differences appeared in primary human DCs: cell death occurred at 72 h post-infection with African strains, while the Asian lineage caused no detectable mortality [93,105]. In human neural progenitors and astrocytes, African strains caused marked deregulation of genes linked to cell cycle, mitosis, and neuronal differentiation, whereas Asian strains preferentially increased TP53 expression, a key regulator of the caspase-3 cell death pathway (>80% vs. 4% for African lineage) [95,96,106]. A complementary experiment showed that TP53 inhibition markedly reduced apoptosis induced by the Asian strain but had a weaker effect on African strain–mediated cytotoxicity, suggesting distinct cell death pathways between the two lineages in human neural progenitor cells (hNPCs) [106].

In cerebral organoids, African strains more strongly suppressed neuronal proliferation and preferentially targeted immature neurons, intermediate progenitors, and astrocytes at the organoid surface. By contrast, Asian strains mainly infected apical progenitors in the ventricular zone and promoted differentiation [107]. Similar lineage-dependent effects were found in human pericytes: the Asian epidemic strain H/PF/13 induced aberrant calcification through BMP2 secretion, a mechanism likely linked to CZS symptoms. In contrast, the African strain MR766, although also stimulating BMP2 in U2OS cells, caused extensive cell death that limited this effect [101].

At tissue barriers, African strains induced greater retinal disruption. In the BBB, however, both lineages preserved overall integrity. Infection nonetheless led to the upregulation of adhesion molecules and tight-junction genes, with occludin, a specific marker of tight junctions, showing stronger expression after infection with African strains [99,100]. Finally, in trophoblast cells, only the African lineage caused severe placental lysis, while Asian strains induced only mild effects [102,103]

Thus, African lineage viruses generally trigger stronger cytopathic effects and acute cell death, whereas Asian strains favor persistence and delayed alterations of cellular pathways (Table 1).

### 4.3. Comparison Regarding Immunity

#### 4.3.1. Induction of IFN

**Figure 3:**
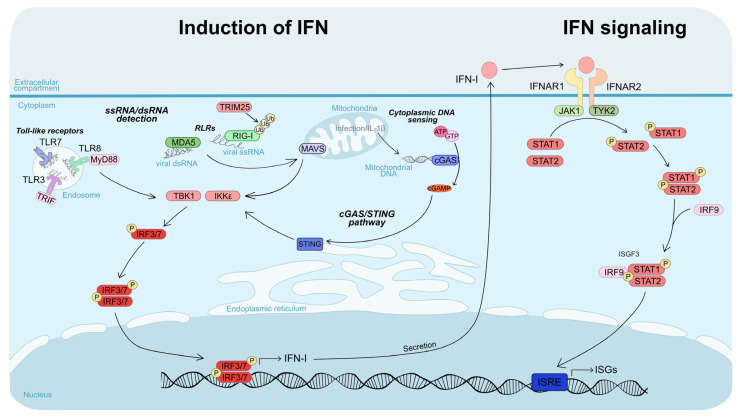
**Schematic representation of the interferon response.** The figure is divided into two parts: the left part illustrates the pathways leading to type I interferon (IFN-I) induction, while the right part depicts the IFN signaling cascade. Type I IFN induction is triggered by the recognition of pathogen-associated molecular patterns (PAMPs) by pattern-recognition receptors (PRRs). During flavivirus infection, viral genomes are primarily sensed by the cytoplasmic RIG-I-like receptors (RLRs), RIG-I and MDA5. Once activated, these sensors signal through the adaptor MAVS located on the outer mitochondrial membrane, leading to activation of the kinases TBK1 and IKKε, which phosphorylate IRF3 and IRF7. The activated transcription factors translocate into the nucleus to induce type I IFN expression. Viral components can also be recognized in endosomes through Toll-like receptor (TLR) signaling. In addition, flavivirus infection can activate the cGAS-STING pathway indirectly, through mitochondrial DNA leakage. Activated cGAS synthesizes the second messenger 2′,3′-cyclic GMP–AMP (cGAMP), which binds to STING at the endoplasmic reticulum, promoting further activation of TBK1 and IKKε. Once secreted by infected cells, type I IFNs bind to their receptor composed of the IFNAR1 and IFNAR2 subunits. This interaction activates the kinases TYK2 and JAK1, which phosphorylate STAT1 and STAT2. The phosphorylated STAT1–STAT2 heterodimers recruit IRF9 to form the ISGF3 complex, which translocates into the nucleus and induces the expression of hundreds of interferon-stimulated genes (ISGs), establishing an antiviral state in both infected and neighboring cells. Created using Inkscape 1.4.2.

Interferons (IFNs) are antiviral cytokines divided into three main types: type I (e.g., IFN-α, IFN-β), type II (IFN-γ), and type III (IFN-λ), each with distinct receptors and functions. IFN-I are central to the antiviral response because they are produced by most nucleated cells, act broadly, and establish the initial antiviral state. Viral recognition by intracellular sensors such as TLRs, RIG-I, MDA5, and cGAS/STING activates the kinases TBK1 and IKKε, which phosphorylate transcription factors IRF3 and IRF7 (Figure 3). These factors then drive IFN-I transcription, and the cytokines are secreted. IFN-I binds to IFNAR receptors on both infected and neighboring cells, activating the JAK1/TYK2 kinases, which phosphorylate STAT proteins. Phosphorylated STAT1/STAT2 associates with IRF9 to form the transcription factor ISGF3, which induces interferon-stimulated genes (ISGs). ISGs limit viral replication, modulate apoptosis, and shape both innate and adaptive immune responses [55,108] (Figure 3).

Whether ZIKV lineages differ in their activation of IFN responses remains unclear, as studies report contrasting results across cell types and experimental models (Table 1). Evidence from some reports suggests that the African lineage can trigger stronger immune activation. For instance, in human DCs and macrophages, African strains induce higher expression of RIG-I, MDA5, and type I/II IFNs, whereas Asian strains can sometimes suppress their expression [96]. Similarly, in BBB cells and primary astrocytes, African strains enhance IL-6 production and markedly upregulate IFN-β transcription [100].

Nonetheless, the Asian lineage has also been shown to elicit robust immune responses under certain conditions. For example, Österlund et al. (2019) reported that an Asian strain induced a more effective antiviral response than the African lineage in human DCs [94]. In line with this, a pre-epidemic Cambodian strain more effectively stimulated ISG expression than the African strain MR766 in hNPCs [106], while an Asian strain from the 2015 Mexican outbreak triggered a strong antiviral response in primary human neural stem cells (hNSCs) [109]. By contrast, African strains have been associated with a delayed induction of antiviral gene in astrocytes [98].

Adding further complexity, some studies indicate that immune activation may be strain-specific rather than strictly lineage-dependent. For instance, infection with an epidemic Brazilian (Asian lineage) strain induced weaker IRF3 nuclear translocation than an African strain or a pre-epidemic Asian strain, reflecting reduced innate immune activation [50]. Furthermore, Xia et al. (2018) identified a mutation in NS1 (A188V), present in both African and post-2013 American epidemic strains, which enhances NS1 binding to TBK1, inhibits its phosphorylation, and reduces IFN-β production [110]. Taken together, these findings suggest that differences in innate immune activation are not solely determined by lineage, but may instead reflect strain-specific adaptations (Table 1). In this context, it is possible that the African lineage originally possessed strong innate immune-modulatory capabilities that were lost and subsequently re-acquired in certain epidemic Asian strains, underscoring the dynamic evolution of ZIKV–host interactions.

#### 4.3.2. Interferon Signaling

African ZIKV strains consistently exhibit greater resistance to IFN-I response compared with Asian lineage viruses (Table 1). Multiple studies have demonstrated that African lineage viruses are less sensitive to IFN-I in both immortalized cell lines (Vero and A549) and primary human monocytes [90,91,111], although the mechanisms underlying this enhanced resistance remained elusive. While ZIKV NS5-mediated degradation of STAT2 is a well-established mechanism for inhibiting JAK/STAT signaling [112], Willard et al. (2017) showed that both African and Asian lineages degrade STAT2 to a similar extent, indicating that this pathway does not account for the observed lineage-specific differences in IFN-I sensitivity [90]. Instead, our team demonstrated, by overexpressing a panel of 30 ISGs, that lineage-specific resistance to ISGs largely explains the enhanced IFN-I resistance of African strains [91]. We hypothesized that more effective compartmentalization of viral replication within replication factories could contribute to the observed broad ISG resistance, although this remains to be experimentally validated [91].

Additional mechanisms may further contribute to lineage-specific differences in IFN-I sensitivity. For instance, Bowen et al. (2017) reported that African and Asian strains differ in their modulation of STAT protein phosphorylation in DCs, with African strains more efficiently inhibiting phosphorylation and thereby limiting downstream ISG expression [93].

#### 4.3.3. Immune Response

Beyond IFN-I signaling, ZIKV lineages exhibit distinct immunomodulatory effects. Infection of CD14^+^ monocytes with African-lineage ZIKV typically induces a classical M1-skewed pro-inflammatory response, characterized by elevated expression of cytokines such as CXCL10 and IL-23A, whereas infection with Asian-lineage ZIKV tends to promote a non-classical M2-skewed immunosuppressive profile, driving IL-10 production and dampening inflammatory responses [113]. Interestingly, pregnancy appears to accentuate these lineage-specific differences: blood from pregnant women infected with Asian-lineage ZIKV shows enhanced M2-skewed immunosuppression, reduced IFN-I signaling, and altered expression of genes associated with pregnancy complications [113]. Conversely, Hernández-Sarmiento et al. (2023) reported that monocytes infected with American strains (e.g., Puerto Rico and Colombia) generally mount stronger pro-inflammatory and IL27-dependent antiviral responses than those infected with African strains (e.g., Nigeria and Dakar) [114]. They further demonstrated that IL27 induces antiviral proteins such as APOBEC3A, ISG15, and Viperin, and that African strains are more resistant to the antiviral effects of this cytokine in monocytes [114]. Collectively, these findings indicate that African and Asian ZIKV lineages differ not only in their sensitivity to IFN-I and ISG-mediated restriction but also in their influence on monocyte polarization and broader immune responses, which may contribute to the distinct pathogenic outcomes observed across lineages. It is worth noting, however, that no clear consensus has yet emerged regarding the monocyte polarization induced by African versus Asian strains (Table 1).

**Table 1:** Comparative in vitro features of African and Asian ZIKV lineages.

Characteristic	Favored Lineage	Reported Difference	References
Replication in cell lines	African	African strains replicate faster and reach higher viral titers	[89,90,91,92]
Replication in primary cells	African	African strains show faster replication kinetics and higher titers	[91,93,94,95,96,97,98,99,100,101,102,103,104]
Cytotoxicity	African	African strains induce stronger cytopathic effects and higher levels of cell death	[89,93,95,96,99,100,101,102,103,105,106,107]
IFN-I response activation	No consensus (African/Asian)	Results vary depending on the cell type and viral strain tested	[50,94,98,100,106,109,110]
IFN-I resistance	African	African strains exhibit enhanced resistance to type I IFN responses	[90,91,93]
Monocyte polarization	No consensus (African/Asian)	Variable outcomes depending on strain and conditions	[113,114]

For clarity and to maintain stylistic consistency with the figures, “Asian” is indicated in red and “African” in blue."

## 5. In Vivo Studies

### 5.1. Murine Model

Murine models have been instrumental in comparing ZIKV lineages, particularly IFN-deficient mice, where impaired IFN-I signaling allows productive infection due to the virus’s inability to efficiently antagonize murine STAT2 [112,115].

Consistent with in vitro observations, African-lineage viruses tend to be more virulent than Asian strains, producing higher viral loads and causing severe tissue damage in organs including the brain, spleen, testes, and eyes [89,99,116] (Table 2).

Infection with African strains leads to rapid viral replication, greater neuronal destruction following intracerebral inoculation, and earlier mortality, while Asian-lineage infections typically result in more prolonged, less acute neurological symptoms [97,117]. The heightened pathogenicity associated with African strains is accompanied by strong inflammatory responses, including elevated cytokines and increased T-cell infiltration, whereas both lineages elicit robust CD8^+^ T-cell activation, with subtle differences in antigenic targeting that may influence the antiviral response [117,118].

Regarding vertical transmission, African strains have been found to cross the placental barrier efficiently in AG129 mice, leading to high rates of spontaneous abortion and severe brain tissue damage with elevated viral RNA in offspring. In comparison, Asian-lineage infections primarily cause reduced fetal head size and umbilical cord thickness [119]. Indeed, infection of pregnant dams with an epidemic Asian strain led to increased expression of osteogenic genes and brain calcifications in pups, which may contribute to microcephaly [101].

In immunocompetent mice, intraperitoneal inoculation is generally non-lethal, whereas intracranial administration results in near-complete mortality with African strains, but limited or no mortality with Asian strains, further highlighting the pronounced pathogenic potential of African lineages [92,120].

Collectively, these studies illustrate that lineage-specific differences in viral replication, tissue tropism, immune activation, and vertical transmission contribute to the distinct disease outcomes observed in murine models (Table 2).

### 5.2. Non-Human Primate Models

Comparative studies of ZIKV lineages in non-human primates remain limited. Investigations into African strains during pregnancy in rhesus macaques have reported that low-dose Senegalese isolates can result in vertical transmission, with virus detected in placental and fetal membranes and infectious particles in amniotic fluid, yet without major fetal pathology [121]. In contrast, higher-dose inocula have been associated with early fetal losses in some animals, suggesting a dose-dependent effect [122]. These findings indicate that African strains circulating in sylvatic environments can pose a risk to primate fetuses, similar to the threat posed by Asian strains in humans.

Direct comparisons of African and Asian strains in pregnant rhesus macaques have suggested that both lineages exhibit similar replication kinetics, immune responses, and limited fetal anomalies, although African strains tend to produce higher viral loads in maternal–fetal interface tissues, particularly in the placenta, fetal membranes, and mesenteric lymph nodes [123]. In non-pregnant cynomolgus macaques, Asian strains have been found to cause prolonged viremia (up to 10 days), with viral persistence in testes, urine, and saliva, whereas the tested African strain failed to establish detectable infection [124]. Similar observations in Indian rhesus macaques using the same African isolate (IbH30656) have been attributed to strain-specific effects, possibly reflecting attenuation due to extensive laboratory passaging, whereas more recent low-passage African isolates (e.g., DAK-AR-41524/1984) display higher virulence [121,125].

Overall, while African strains may replicate more efficiently in maternal–fetal tissues, some laboratory-passaged isolates seem attenuated in macaques (Table 2). These results must be interpreted cautiously, given that non-human primates constitute natural ZIKV hosts and their symptomatology is shaped by long-term host–pathogen coevolution.

### 5.3. Other Models

Although less commonly employed, avian models have been investigated as vertebrate systems for ZIKV infection. In chicken embryos, infection with an African strain has been reported to cause higher mortality than an Asian strain, even at lower inoculum doses, and is associated with increased tissue viral loads and a higher proportion of infected embryos [90] (Table 2). More recently, ZIKV infection models have also been developed in zebrafish [126,127], but to date no direct comparisons between African and Asian ZIKV lineages have been performed in this system.

## 6. Vector Competence

Despite some variation across mosquito populations, studies consistently report higher transmission potential for African ZIKV strains compared with Asian strains. *Aedes aegypti* and *Aedes albopictus* from New Caledonia have exhibited greater competence for African strains, with infection rates exceeding 37% at 9 days post-infection (dpi) compared with less than 10% for Asian strains, and with faster infection kinetics [128]. Viral detection in saliva occurred as early as 7 dpi for African strains, whereas it was typically observed around 14 dpi for Asian strains [119]. Similar patterns have been observed in Brazilian *A. albopictus*, which transmitted African but not Asian strains effectively [129], and in *A. aegypti* populations from Mexico, Florida, Colombia, El Salvador, Texas, the Dominican Republic and Cambodia [89,92,119,130,131]. Only one study reported higher infection of Mexican *A. aegypti* by Asian strains, but saliva transmission levels were comparable for both lineages [90].

African strains also demonstrate enhanced viral dissemination within mosquitoes, spreading efficiently from the midgut to the legs, wings, and salivary glands, a critical step in vector competence, whereas Asian strains often exhibit more limited spread, with some intra-lineage differences [89,131] (Table 2).

At the molecular level, a residue in NS1 (A188V) under positive selection has been identified as a transmission determinant. Valine at this position, present in African strains but absent in pre-epidemic Asian strains, enhances infectivity and transmission by *A. aegypti*, partly explaining the superior vector competence of African ZIKV [51,59,132]. Collectively, these data underscore the high epidemic potential of African ZIKV strains.

**Table 2:** Comparative in vivo outcomes of African and Asian ZIKV lineages.

Host Model	Characteristic	Favored Lineage	Reported Differences	References
Mouse	Physiological alterations and lethality in adults	African	African strains cause greater tissue damage and higher lethality in adult mice	[89,92,97,99,116,117,118,120]
	Fetal damage	African	African strains induce spontaneous abortion and more severe brain damage in pups	[101,119]
Avian	Lethality	African	African strains increase mortality in chicken embryos	[90]
Non-human primates	Infection level	No consensus (African/Asian)	Infection levels vary depending on viral strain and primate species	[123,124,125]
	Fetal damage	African	African strains result in higher viral loads at the maternal–fetal interface	[121,122,123]
Mosquito vectors	Infection	African	African strains generally exhibit higher vector competence	[51,89,90,92,119,128,129,130,131]
	Dissemination	African		
	Transmission	African		

For clarity and to maintain stylistic consistency with the figures, “Asian” is indicated in red and “African” in blue.

## 7. Exceptions to the General Trend

Although African ZIKV strains generally exhibit higher replication and virulence, a limited number of studies report exceptions, suggesting that under certain conditions, Asian strains may replicate more efficiently or display greater pathogenicity. In human endothelial cells derived from umbilical, aortic, coronary, and saphenous veins, South American Asian strains have been observed to replicate to higher levels, form larger plaques, and induce more cell death than African strains [133]. In addition, one study reported that human monocytes may be more susceptible to infection with Asian strains compared with African strains [114].

Evidence from neurotropism studies further highlights deviations from the typical pattern. In cerebral organoids, the extent of brain disruption was comparable between lineages, while MR766 generated larger neurospheres than a Brazilian Asian strain, suggesting relatively lower impairment of neural progenitors, although neuronal mortality did not differ significantly [134,135].

In vivo, Ifnar1−/− mice infected with the Polynesian Asian strain H/PF/2013 exhibited 100% lethality, whereas survival ranged from 20 to 60% with African MR766, contrasting with most murine data [136].

These observations indicate that differences in replication and virulence between African and Asian ZIKV lineages are influenced by cell type, experimental model, and specific viral strains, emphasizing the complexity and context-dependence of ZIKV infection dynamics.

## 8. Molecular Basis of Lineage-Specific Traits

### 8.1. Mutations

#### 8.1.1. Structural Proteins

Several lineage-specific mutations in ZIKV structural proteins have been identified as critical determinants of pathogenicity and neurovirulence (Table 3).

In the E protein, the VNDT glycosylation motif, commonly found in most Asian strains, particularly post-2015 American epidemic isolates, but inconsistently observed in African strains, is critical for neuroinvasion and is linked to more severe central nervous system infections in murine models [137]. This motif introduces an asparagine (N) residue at position 154, which undergoes glycosylation, a modification that facilitates viral entry. Additionally, residues at positions 151, 156, and 158 play key roles in the pH-induced conformational changes of the E protein. These four sites vary between lineages, with most African strains being non-glycosylated, in contrast to Asian strains [138]. During endocytosis, the E protein also undergoes protonation (addition of a hydrogen ion) at several sites, notably at the H158 residue within the glycan loop. This protonation triggers conformational rearrangements essential for forming the fusion pore required for viral entry. Interestingly, African strains appear to lack this protonation site, along with the glycosylation at N154. The absence of these two features suggests a potentially reduced entry efficiency, although this has not yet been confirmed in vitro [138]. Finally, the E-V763M substitution observed in Asian strains has been shown to enhance viral replication, neurovirulence in neonatal mice, and maternal–fetal transmission [139].

In the prM protein, the S139N mutation, characteristic of pandemic Asian strains, increases virulence in hNPCs and neonatal mice. Site-directed mutagenesis has shown that introducing S139N into a 2010 Cambodian strain raised neonatal mortality, whereas reversing it to N139S in a 2016 epidemic strain reduced virulence [140]. Its effect on maternal–fetal transmission remains debated, as some studies report minimal impact and African strains lacking S139N can still induce severe fetal neuronal damage, suggesting potential redundancy [141]. S139N has also been associated with increased neuronal apoptosis, accelerated microglial activation, and reduced CD8+ T-cell responses [142].

African strains carry an E21K mutation in prM, which is a major determinant of neurovirulence and neuroinvasion. Introducing this mutation into an Asian strain increased neuronal replication, neurological damage, and antiviral immune activation, whereas reversing it to K21E in African strains reduced neurovirulence in mice [143]

Additional structural protein mutations, including C-A109T and prM-A1V, have been proposed as potentially advantageous, although their phenotypic consequences remain to be determined [132].

#### 8.1.2. Non-Structural Proteins

Mutations in ZIKV non-structural proteins also contribute to viral virulence, immune evasion, and vector competence (Table 3). Among the most studied is the A188V substitution in NS1, present in African strains and post-2012 epidemic Asian lineages. This mutation confers a selective advantage. While early Asian strains had reverted from valine (V) to alanine (A), recent epidemic lineages have reacquired valine, restoring the ancestral amino acid at this position [59]. NS1-A188V enhances interferon resistance, facilitating host infection [110] and promotes transmission in *Aedes aegypti* by increasing NS1 secretion and vector infectivity [51]. Co-infection competition experiments further demonstrate that NS1-A188V is a dominant determinant of viral fitness [132].

Other non-structural protein mutations, such as NS5-V872M, have also been identified as potentially advantageous, although their functional consequences remain to be elucidated [132].

#### 8.1.3. UTRs

ZIKV UTRs also play a key role in viral fitness. In the 5′UTR, the recently identified uORF differs between lineages: African strains carry a single uORF, whereas Asian strains contain two (uORF1 and uORF2). Mutagenesis and viral competition experiments in U251 glioblastoma cells revealed that deletion of uORF1 in an Asian strain, or its replacement with the African uORF, enhanced viral replication and translation of the main ORF, whereas inactivation of uORF2 had no effect [34].

In cerebral organoids, however, the African uORF or deletion of uORF1 reduced neuronal infection, indicating that uORF1 promotes viral dissemination in neural tissue. Mechanistically, uORF1 interacts with intermediate filaments, inducing cytoskeletal reorganization around viral factories, whereas the African uORF preserves normal vimentin localization. This sequence does not affect viral infectivity in mosquitoes, suggesting a host-specific function in mammals [34].

In the 3′UTR, Asian strains harbor thirteen lineage-specific mutations absent in African strains, which stabilize stem-loop structures. These changes may influence sfRNA production, thereby modulating resistance to host antiviral pathways [63]. Full-length RNA structural analyses further indicate lineage-specific differences that likely impact viral fitness [144].

**Table 3:** Key divergent mutations between ZIKV lineages and their functional effects.

Protein	Mutation	Reported Effect	Predominant Lineage	References
E	VNDT motif	↑ Lethality and neuroinvasion	Asian Some African strains	[137]
	V763M	↑ Replication, ↑ maternal–fetal transmission, ↑ neurovirulence in mouse pups	Asian (post-2015)	[139]
prM	E21K	Neurovirulence and neuroinvasion factor	African	[143]
	S139N	Neurovirulence factor (although African strains without this mutation cause greater neuronal damage)	Epidemic Asian strains	[140,141,142]
NS1	A188V	↑ Type I IFN resistance, ↑ secretion, ↑ mosquito transmission	African, Asian (post-2012)	[51,110,132]
5′UTR	Unique uORF	↑ Replication, ↑ translation	African	[34]
	uORF1 and uORF2	uORF1 linked to neuronal dissemination	Asian	

↑: increase. For clarity and to maintain stylistic consistency with the figures, “Asian” is indicated in red and “African” in blue.

### 8.2. Codon Usage and Its Implications for Lineage-Specific Pathogenicity

Codon usage bias reflects the non-random preference for synonymous codons, influencing translation efficiency and accuracy according to tRNA abundance [145]. In the context of viral infections, adaptation to host codon usage can play a key role during host switching, allowing the virus to fine-tune interactions with the new organism. Such adaptation serves as an evolutionary indicator of host-switching processes [146].

Significant differences exist between African and Asian ZIKV lineages in terms of codon adaptation, which may relate to differences in pathogenicity. The Asian lineage shows enhanced adaptation of NS1 to human and *Aedes aegypti* codon usage, with additional non-structural proteins (NS2A, NS2B, NS4B) better adapted to mosquitoes but not humans. Conversely, the African lineage shows superior adaptation to humans for E, NS4B, NS5 and to mosquitoes for E and NS5. Functional studies in human neural progenitor cells (hNPCs) confirmed higher expression of NS1, NS2B, and E proteins in Asian strains, supporting a link between codon bias, translation efficiency, and viral fitness in human cells [146].

However, codon adaptation in the Asian lineage likely reflects post-emergence expansion rather than a driver of emergence, as NS1 codon usage progressively increased during its spread across the Pacific and Americas [146]. These codon usage biases may influence viral replication and immune evasion, with NS1 playing a critical role in immune evasion and NS5 affecting both replication and immune modulation [145,146]. Overall, codon adaptation appears to represent a consequence of viral diversification rather than a cause of emergence, although it may contribute to lineage-specific differences in protein expression and immune interactions (Table 4).

## 9. Conclusions and Perspectives

Comparative analyses of African and Asian ZIKV lineages highlight the complexity of viral emergence and underscore the need to consider multiple determinants when understanding epidemic dynamics. Although the African lineage exhibits higher intrinsic fitness [50,90,93,95,96,98,102,103,107,109], enhanced resistance to innate immunity [90,91,93,110,111,114], increased neurovirulence [89,90,97,116,117,119,120,136,141,147], and superior vector transmissibility [51,119,130,131,148], it was the Asian lineage that drove major human epidemics in the 21st century. This paradox illustrates that viral genetic advantages alone cannot explain emergence, which instead results from a complex interaction of viral, host, vector, immune, ecological and socio-demographic factors.

Historical underreporting and limited circulation may partly explain the apparent absence of large African outbreaks, as incomplete surveillance, misdiagnosis as other pathogens, and outcomes such as early fetal loss rather than overt congenital malformations likely obscured severe infections [15,119]. In contrast, the 2015–2016 emergence in immunologically naïve populations in the Americas coincided with intense scientific and media attention, amplifying the detection of complications. Nevertheless, seroprevalence studies indicate genuinely limited ZIKV circulation in Africa, with continental averages around 8.4% [14,149,150,151], indicating that sustained human-to-human transmission was rare. Given this context, why did the superior replication efficiency, cytopathogenicity, and transmissibility of African strains not translate into higher epidemic potential? Their relatively limited epidemiological impact suggests that these intrinsic advantages are counterbalanced by additional factors in natural settings. Several non-exclusive hypotheses can help reconcile this discrepancy.

One key factor may be the trade-off between virulence and transmission. The pronounced cytopathic effect of African strains can cause rapid host cell destruction, potentially shortening the duration of viremia and narrowing the window for mosquito transmission. In addition, high virulence may restrict host mobility, further limiting opportunities for viral spread. In contrast, Asian strains, by inducing milder cellular damage, may sustain longer infections, maintain host mobility, and thereby facilitate wider transmission [152,153,154,155,156]. Modeling studies suggest that moderate virulence represents the most advantageous strategy for viral emergence, as prolonged infections increase the likelihood of adaptive mutations [157]. Low to moderate virulence, possibly coupled with a founder effect, followed by structural and molecular changes in the Asian lineage, such as E-N154 glycosylation, prM-S139N, and NS1-A188V, may have further enhanced neurotropism, interferon evasion, and transmission efficiency in humans, ultimately contributing to the epidemic success of the Asian lineage [51,110,132,137,138,140].

The infection dynamics associated with moderate virulence likely also contributed to these divergent outcomes. The persistence of Asian strains, particularly in immune-privileged sites such as genital tissues, may have facilitated secondary transmission routes, including sexual spread. Mathematical modeling studies have shown that sexual transmission can amplify both the magnitude and duration of outbreaks [124,158,159]. Although African strains can experimentally infect non-human primates through mucosal exposure [160], sexual transmission in natural settings remains uncertain. In this context, social and behavioral factors, including patterns of sexual contact and exposure to mosquito vectors, likely modulate the relative contribution of these transmission routes, underscoring the importance of integrating social science perspectives into epidemiological analyses.

Host immune responses likely also played a critical role in shaping these divergent epidemic outcomes. In Asia, hyperendemic DENV circulation with seroprevalence levels of 50 to 75% [161] may have created an immune environment that favored ZIKV through ADE [162,163]. In Africa, lower DENV exposure (0–35%) may have limited such cross-reactive effects. [161]. Moreover, Asian strains appear to induce a more regulatory immune response, whereas African strains elicit stronger pro-inflammatory profiles [113,117], potentially accelerating viral clearance and limiting transmission.

Host genetic variability adds yet another layer of complexity. A striking example is a study of dizygotic twins exposed to the same ZIKV strain in utero, where one child developed disease and the other did not. This demonstrates that the host background can determine neuronal susceptibility [65,164]. Genetic differences between populations may have influenced viral clearance and disease severity. A notable example of that is the IFNL4 polymorphism, which encodes interferon-λ4: ΔG allele, that allows production of the functional protein, is more frequent in populations of African ancestry, whereas the TT allele, predominant in European and Asian populations, prevents IFN-λ4 expression and is associated with more efficient clearance of Hepatitis C Virus [165,166].

Finally, environmental and anthropogenic drivers strongly influence epidemic risk. Seasonal temperature and humidity affect mosquito density and behavior, shaping transmission windows and outbreak magnitude [65,167,168,169]. Extreme climate events, such as the 2015–2016 El Niño, facilitated the epidemic in the Americas [170]. Human-driven ecosystem changes including deforestation, urbanization, water management, agricultural expansion, global trade, and air travel further increase contact with vectors and promote viral spread [171,172,173]. Demographic trends also raise transmission risk [174]. Addressing these challenges requires integrated approaches that combine environmental governance, ecosystem restoration, and population-level interventions such as education [175].

An additional question raised by recent observations is why large-scale ZIKV outbreaks have virtually disappeared since 2017. Several non-exclusive hypotheses can be considered: (i) the establishment of strong herd immunity following the major epidemics of 2015–2017, which markedly reduced the pool of susceptible hosts; (ii) a decline in vector competence within post-epidemic *Aedes aegypti* populations; (iii) more effective vector control measures and increased public awareness; and (iv) possible viral interference or ecological competition with other co-circulating arboviruses such as DENV and CHIKV. Although such interference appears limited in *Aedes* mosquitoes, since co-infections with ZIKV and DENV or CHIKV generally do not result in strong viral competition within the vector [176,177], it could potentially occur in vertebrate hosts where inter-arboviral interactions may influence infection dynamics. In addition, climatic fluctuations likely contribute to temporal and spatial variations in mosquito abundance and activity, further shaping transmission cycles. Despite the apparent disappearance of major epidemics, silent, low-level ZIKV circulation likely persists, underscoring the importance of maintaining continuous serological and genomic surveillance to detect potential re-emergence as population immunity gradually wanes.

In summary, the divergent epidemic patterns of African and Asian ZIKV lineages reflect a complex interplay between viral virulence, transmissibility, host immunity, and ecological context. Understanding this multifactorial equilibrium is crucial not only to elucidate past epidemic dynamics but also to anticipate and mitigate future arboviral emergence [65,178].

## Figures and Tables

**Table 4:** **Codon usage bias of ZIKV proteins in human and mosquito hosts.** Data adapted from [146].

Protein	Codon Bias in Humans	Codon Bias in *A. aegypti*
C	None	None
PrM	Asian	African
E	African	African
NS1	Asian	Asian
NS2A	None	Asian
NS2B	African	Asian
NS3	African	African
NS4A	None	Asian
NS4B	African	None
NS5	African	African

For clarity and to maintain stylistic consistency with the figures, “Asian” is indicated in red and “African” in blue.

## Data Availability

No new data were created or analyzed in this study. Data sharing is not applicable to this article.

## Abbreviations

The following abbreviations are used in this manuscript:

ADE	antibody-dependent enhancement
BBB	blood–brain barrier
C	capsid protein
CZS	congenital Zika syndrome
DCs	dendritic cells
DENV	dengue virus
E	envelope protein
hNPCs	human neural progenitor cells
hNSCs	human neural stem cells
IFN	interferon
ISG	interferon-stimulated gene
JEV	Japanese encephalitis virus
M	membrane protein
MTase	methyltransferase
NC	nucleocapsid
ORF	open reading frame
prM	precursor membrane protein
PHEIC	Public Health Emergency of International Concern
RdRp	RNA-dependent RNA polymerase
sfRNA	subgenomic flaviviral RNA
uORF	upstream open reading frame
WHO	World Health Organization
WNV	West Nile virus
YFV	yellow fever virus
ZIKV	Zika virus
